# Editorial: Untangling energy metabolism in skeletal muscle: From physiology to pharmacology

**DOI:** 10.3389/fphys.2022.1113860

**Published:** 2022-12-14

**Authors:** Sergej Pirkmajer, Pablo M. Garcia-Roves, Arild C. Rustan, Alexander V. Chibalin

**Affiliations:** ^1^ Institute of Pathophysiology, Faculty of Medicine, University of Ljubljana, Ljubljana, Slovenia; ^2^ Department of Physiological Sciences, School of Medicine, University of Barcelona, Barcelona, Spain; ^3^ Section for Pharmacology and Pharmaceutical Biosciences, Department of Pharmacy, University of Oslo, Oslo, Norway; ^4^ Department of Molecular Medicine and Surgery, Section of Integrative Physiology, Karolinska Institutet, Stockholm, Sweden; ^5^ National Research Tomsk State University, Tomsk, Russia

**Keywords:** skeletal muscle, energy metabolism, AMPK, myosin, angiotensin-converting enzyme (ACE), metformin, obesity, double-stranded RNA-activated protein kinase

In 1957 Albert Szent-Györgyi stated: “The problem is: how does energy drive life? How does it move the living machine? This is one of the most basic problems of biology and, at present, there is no answer to it” (Szent-Györgyi, 1957). While molecular underpinnings of energy production as well as movement have been uncovered since Szent-Györgyi pointed to the then glaring gap in understanding of fundamental biology, investigation of intimate links between energy metabolism and generation of movement has lost none of its relevance. As highlighted in this Research Topic of Frontiers of Physiology and Frontiers in Pharmacology, skeletal muscles, the specialized molecular machines that convert chemical energy into movement, remain firmly in focus of intense research efforts to untangle molecular mechanisms underlying health and disease.

When Szent-Györgyi and Ilona Banga showed that threads of myosin, a major molecular motor, start to contract in the presence of ATP (Banga and Szent-Györgyi, 1942; Szent-Györgyi, 1942; Rall, 2018), the diversity of myosins could hardly be predicted. Lehka et al. now report that myosin VI, an unconventional myosin that is not part of contractile apparatus, is important for protein kinase A (PKA) signalling in skeletal muscle. In the current study they built on their previous findings that myosin VI interacts with A-kinase anchoring protein 9 (Karolczak et al., 2015), a scaffold for PKA and proteins that regulate its function, and showed that myosin VI was particularly important for PKA signalling in skeletal muscles of newborn mice, the effect being more pronounced in the slow-twitch soleus muscle than in the fast-twitch gastrocnemius muscle. The study raises an interesting possibility that expression of myosin VI is linked to metabolic properties of skeletal muscle and provides the basis for future investigations into age- and fiber type-dependent roles of this unconventional molecular motor in skeletal muscle.

Aside from physiology, three papers included in this Research Topic address various aspects of skeletal muscle pathophysiology, focusing especially on obesity and insulin resistance. In the first paper, Eo and Valentine investigated mechanisms by which increased plasma concentrations of fatty acids may lead to insulin resistance and endoplasmic (ER) reticulum stress in skeletal muscle. Specifically, by extending their previous work (Eo et al., 2020) they provide evidence supporting the role of double-stranded RNA-activated protein kinase (PKR) in palmitate-induced ER stress and insulin resistance. Using C2C12 myotubes they showed that imoxin, an inhibitor of PKR, alleviated ER stress and improved insulin-induced signalling responses as well as glucose uptake in palmitate-treated myotubes, consistent with the idea that PKR might be useful as therapeutic target in insulin resistance and obesity (Nakamura et al., 2014).

The second paper, by Katare et al., examined how obesity affected metabolic properties of skeletal muscle by assessing metabolism in primary human myotubes, a particularly useful experimental model because cultured myotubes maintain various metabolic characteristics of donor tissue (Aas et al., 2013). Proteomic analysis revealed that glycolytic, apoptotic, and hypoxia pathways were upregulated in cultured myotubes from individuals with BMI >30 kg/m^2^. Furthermore, while having increased uptake of substrates, including fatty acids and glucose, myotubes from individuals with obesity displayed lower lipid oxidation and increased glucose oxidation. This result indicates not only a switch in substrate preference, but also that increased capacity for uptake of substrates is not necessarily paralleled by increased capacity to oxidize them, consistent with the observation that obesity is associated with intracellular accumulation of lipids in skeletal muscle, which likely contributes to its metabolic dysfunction (Gilbert, 2021).

The third paper, an *in vivo* study by Flück et al., asked whether signalling response of skeletal muscle to exercise was altered in homozygous carriers of a D allele of angiotensin converting enzyme (ACE). ACE, an essential component of the renin-angiotensin-aldosterone system, which catalyses the conversion of angiotensin I to angiotensin II, has a central role in long-term regulation of arterial pressure. Obesity, type 2 diabetes, and arterial hypertension are thought to have common pathophysiological underpinnings, which would explain why these conditions frequently co-exist as part of the metabolic syndrome. Importantly, both metabolic and cardiovascular diseases belong to a cluster of disorders, appropriately dubbed the diseasome of physical inactivity (Pedersen, 2009), which are promoted by a physically inactive life style. In the current study, ACE DD genotype modulated insulin signalling in response to exercise, which might contribute to increased risk of type 2 diabetes in carriers of the DD genotype (Feng et al., 2002).

Untangling regulation of energy metabolism in skeletal muscles under physiological conditions and its dysfunction under pathophysiological conditions is a conduit to uncovering new potential pharmacological targets, a starting point for development of new pharmacotherapies for metabolic disorders, such as insulin resistance and type 2 diabetes. Among the potential entry points for pharmacological modulation of skeletal muscle metabolism, AMP-activated protein kinase (AMPK), a cellular energy sensor, is particularly promising. Appropriately, pharmacology of AMPK as well as its physiological and pathophysiological significance in skeletal muscle is reviewed in detail by Yan et al. as part of this Research Topic. While new pharmacological AMPK activators are actively being sought, mechanisms of action of metformin, an AMPK activator and the most widely used oral anti-diabetic drug, have not been completely resolved despite decades of use in the clinic. As discussed by Pavlovic et al. in the final paper of this Research Topic, inhibition of the mitochondrial complex I leading to energy stress and activation of AMPK, the traditional explanation of metformin action, had previously been confirmed in cell-based models using high micromolar or millimolar concentrations of metformin, but not if metformin was used in therapeutic concentrations, which are typically well below 50 μM. Pavlovic et al., who revisited this dilemma using C2C12 myotubes and high resolution respirometry, provide convincing evidence supporting the view that metabolic effects of metformin, such as those described in classical study on cultured human myotubes by Sarabia et al. (1992), do not depend on inhibition of complex I or activation of AMPK.

In summary, this Research Topic of six papers brings new mechanistic insights into workings of energy metabolism in skeletal muscle and opens new important questions for future research.
